# Erratum

**DOI:** 10.5935/abc.20190019

**Published:** 2019-02

**January issue of 2019, vol. 112(1), pages 67-75**

In “Sex-Related Effects of Prenatal Stress on Region-Specific Expression of Monoamine Oxidase A and B Adrenergic Receptors in Rat Hearts”, consider Tanja Jevdjovic as the correct form for the name of the author Tanja Jevjdovic.

**January issue of 2019, vol. 112(1), pages 91-103**

In “Arrhythmogenic Right Ventricular Cardiomyopathy/Dysplasia (ARVC/D) - What We Have Learned after 40 Years of the Diagnosis of This Clinical Entity”, table 1 of the Portuguese version, item “5. Arritmias”, consider correct for the phrase “TV não sustentada ou sustentada com morfologia tipo BRD e eixo superior” BRE in place of BRD.

